# High prevalence of somatisation in ICD-11 complex PTSD: A cross sectional cohort study

**DOI:** 10.1016/j.jpsychores.2021.110574

**Published:** 2021-09

**Authors:** Laurence Astill Wright, Neil P. Roberts, Catrin Lewis, Natalie Simon, Philip Hyland, Grace W.K. Ho, Eoin McElroy, Jonathan I. Bisson

**Affiliations:** a Division of Psychological Medicine and Clinical Neurosciences, Cardiff University School of Medicine, UK; b Cardiff & Vale University Health Board, UK; c School of Psychology, Maynooth University, Kildare, Ireland; d Department of Neuroscience, Psychology and Behaviour, University of Leicester, UK; e School of Nursing, The Hong Kong Polytechnic University, Hong Kong

**Keywords:** PTSD, CPTSD, Somatisation

## Abstract

**Background:**

While research demonstrates that somatisation is highly correlated with post-traumatic stress disorder (PTSD), the relationship between International Classification of Diseases 11th edition (ICD-11) PTSD, complex PTSD (CPTSD) and somatisation has not previously been determined.

**Objective:**

To determine the relationship between frequency and severity of somatisation and ICD-11 PTSD/CPTSD.

**Method:**

This cross-sectional study included 222 individuals recruited to the National Centre for Mental Health (NCMH) PTSD cohort. We assessed rates of Patient Health Questionnaire 15 (PHQ-15) somatisation stratified by ICD-11 PTSD/CPTSD status. Path analysis was used to explore the relationship between PTSD/CPTSD and somatisation, including number of traumatic events, age, and gender as controls.

**Results:**

70% (58/83) of individuals with CPTSD had high PHQ-15 somatisation symptom severity compared with 48% (12/25) of those with PTSD (chi-square: 95.1, *p* value <0.001). Path analysis demonstrated that core PTSD symptoms and not disturbances in self organisation (DSO) symptoms were associated with somatisation (unstandardised coefficients: 0.616 (*p*-value 0.017) and − 0.012 (*p*-value 0.962) respectively.

**Conclusions:**

Individuals with CPTSD have higher somatisation than those with PTSD. The core features of PTSD, not the DSO, characteristic of CPTSD, were associated with somatisation.

## Highlights

• People with CPTSD had a higher prevalence of co-morbid somatisation.
• People with CPTSD had a worse somatisation symptom severity.
• The core features of PTSD predicted somatisation.

## 1 Background

The 11th edition of the International Classification of Diseases (ICD-11) [1] identifies Complex Post-traumatic Stress Disorder (CPTSD) as a distinct entity, separate to Post-traumatic Stress Disorder (PTSD). ICD-11 PTSD is primarily a disorder of fear and anxiety defined by the triad of re-experiencing, avoidance and hyperarousal experienced after a traumatic event. CPTSD is characterised by the co-occurrence of these core PTSD symptoms and a group of symptoms known as ‘disturbances in self organisation’ (DSO) which include affective dysregulation, negative self-concept, and disturbed relationships [2]. These DSO symptoms are qualitatively distinct from the core symptoms of PTSD and the DSO symptoms identify the chronic psychological changes which typically result from early or repeated trauma exposure [[3], [4], [5]].

PTSD is commonly associated with depression, substance abuse [6], coronary heart disease [7], type 2 diabetes, autoimmune disease [8], serious infective illness [9] as well as chronic physical symptoms [10]. Compared to those without PTSD, people with PTSD report more gastrointestinal and cardiac problems, along with musculoskeletal pain and general health complaints and worse physical health-related quality of life [11]. Between 50 and 80% of people with PTSD have chronic physical symptoms (long-lasting abnormal bodily sensations) [12] and 9.7% of those with chronic physical symptoms have PTSD [11,13]. This comorbidity of PTSD and physical symptoms results in greater disability, worse severity of symptoms, worse prognosis and lower treatment engagement [12]. This high prevalence combined with poorer outcomes suggests common aetiological mechanisms.

Physicians have traditionally clustered physical symptoms that cannot be fully medically explained into conditions such as irritable bowel syndrome (IBS), chronic fatigue syndrome, temperomandibular joint pain and fibromyalgia [10]. Where there is no clear organic cause for such distressing somatic complaints, psychological and social factors may be exerting a significant influence [14]. The Diagnostic and Statistical Manual of Mental Disorders, Fifth Edition (DSM-5) [15] encompasses both medically explained and medically unexplained symptoms (MUS) as somatic symptom disorders (SSD) and requires the presence of a distressing physical health complaint, in association with excessive concern, preoccupation or anxiety with the somatic symptom that may take up a large amount of time or energy [10]. It is sometimes not the symptoms themselves that define SSD but the way they are interpreted [15]. This suggests a large psychobehavioural overlay in a condition with an uncertain physical pathophysiology [16], with considerable personal and societal cost [17].

To our knowledge, no studies have investigated somatic symptom severity in those diagnosed with ICD-11 CPTSD. Consideration of previous conceptualisations of CPTSD and associations with somatisation, however, allows us to hypothesise about the relationship. CPTSD was previously conceptualised through disorders of extreme stress not otherwise specified (DESNOS) [18], a diagnosis of which required somatisation as one of six criteria. Previous studies suggested that the prevalence of DESNOS in people with somatisation disorder is high (current: 35.7%; lifetime: 50%) [19,20], with multiple factors associated with the development of somatisation and DESNOS, such as childhood physical/sexual abuse [19], feelings of guilt, loneliness, mistrust, depressive symptoms [21], affect dysregulation [22] and dissociation [23]. Thus it is possible that for similar reasons to DESNOS, individuals with CPTSD will also have high rates of somatisation.

This cross-sectional cohort study aimed to determine if Patient Health Questionnaire (PHQ-15) somatisation severity was more strongly associated with ICD-11 PTSD or CPTSD and to assess if DSO or core PTSD features were more strongly associated with PHQ-15 somatisation (using covariates of age, gender, and number of previous traumatic events). Based on the previous associations with DESNOS, we anticipated that the strength of association would be greater with DSOs rather than core PTSD symptoms.

## 2 Methods

### 2.1 Data source

Data were obtained from the National Centre for Mental Health (NCMH), a Welsh Government-funded Research Centre [27] that investigates psychiatric disorders across the lifespan. This NCMH cross-sectional cohort study was granted ethical approval by Wales Research Ethics Committee 2. The Centre is operated by Cardiff, Swansea, and Bangor Universities, in partnership with the National Health Service (NHS) across Wales and England. The cohort of participant volunteers primarily included individuals who have experienced a mental disorder, but some individuals without such a history have also been recruited into the cohort. Participants were recruited using a variety of systematic approaches in primary and secondary health care services, including (a) the identification of potential participants by clinical care teams; and (b) screening of clinical notes. Non-systematic recruitment approaches included advertising in local/national media and engaging third-sector organizations to support and promote the research. All adult participants with sufficient mental capacity provided written informed consent to participate. Trained researchers then administered a standardised interview assessment to ascertain sociodemographic information and details related to the participant's history of mental illness. Participants were given a pack of validated self-report questionnaires to complete and return to the research team after the initial assessment. This provided information on 349 participants.

### 2.2 Analysis sample

Participants were 16 years of age or older, reporting exposure to a traumatic event fulfilling requirements for a diagnosis of PTSD and CPTSD under DSM-5 and ICD-11. Participants self-reported a current/historical diagnosis of PTSD or reported having experienced a traumatic event which satisfies the Trauma Screening Questionnaire gatekeeper criterion, clarifying that the participant has been exposed to a traumatic event that would satisfy the DSM-5 A criterion [15,28]. Individuals who were unable to read and write in English were excluded, as were people who had recently been a mental health inpatient or were in frequent contact with a crisis related intensive home treatment team, due to the risk of exacerbating psychological distress. This provided information on 349 participants, of whom 222 completed the International Trauma Questionnaire (ITQ).

### 2.3 Measures

We used a modified version of the life events checklist for DSM-5 (LEC-5) to screen for potentially traumatic events over a participant's lifetime according to DSM-4 criteria [29]. The LEC is a well validated measure using a 5-point nominal scale to measure different types of exposure to potentially traumatising events (1 = happened to me, 2 = witnessed it, 3 = learned about it, 4 = not sure, 5 = doesn't apply). Internal consistency is very good (Cronbach's alpha = 0.94 [29]). A participant was considered exposed to a traumatic event if they reported either direct exposure to, witnessing or hearing about (only in the event of sudden violent death) a single LEC item, and the number of these exposures was summed to give a total LEC score. The modification was to include two additional items assessing exposure to childhood physical abuse and childhood sexual abuse or molestation.

We used the ITQ to determine probable ICD-11 diagnoses of PTSD and CPTSD. The ITQ is a self-report measure using 12 symptom indicators measured on a five-point Likert scale [30]. Symptom rating ranged from ‘not at all (0)’ to ‘extremely (4)’. Probable PTSD diagnosis requires the presence of one of the two symptoms from each of the three core PTSD clusters (re-experiencing, avoidance and a persistent sense of threat). Probable CPTSD diagnosis requires the presence of one of the two symptoms from each of the three DSO symptom clusters (affective dysregulation, negative self-concept and disturbed relationships), in addition to the fulfilment of PTSD criteria [30]. Both a PTSD and CPTSD diagnosis also necessitate an impairment in functioning due to these symptoms. Composite reliability testing demonstrates the ITQ has excellent internal reliability, with composite reliability findings of 0.86–0.96 depending on which sub scale is investigated [31]. The ITQ has been shown to distinguish between core PTSD and DSO symptomatology in several studies [[30], [31], [32]]. Validation of the ITQ suggests the ICD-11 criteria for PTSD/CPTSD are stricter than DSM-5 requirements, identifying a subset of individuals who qualify for a diagnosis of PTSD under DSM-5, but not for PTSD or CPTSD under ICD-11 [31].

We used the PHQ-15 to measure self-reported somatic symptom severity over the past 7 days. Each of the fifteen items on the PHQ-15 is rated on a 3-point Likert scale ranging from ‘not bothered at all’ to ‘bothered a lot’ [33]. The total score is continuous and can range from 0 to 30, it can also provide a categorical measure of somatic symptom severity. A total score of 0–4 indicates minimal somatic symptom severity, 5–9 indicates low, 10–14 indicates medium, and 15–30 indicates high symptom severity [33]. The PHQ-15 is well validated and has a sensitivity of 78% and a specificity of 71% for a DSM-IV diagnosis of somatoform disorder in a primary care setting using a cutoff of 3 or more severe somatic symptoms over the preceding 4 weeks [34]. Furthermore, internal consistency is good (Cronbach's alpha = 0.80) [33].

### 2.4 Analyses

Demographic and frequency analyses were performed using the Statistical Package for Social Science [35]. We used Mplus (Version 8.3 [36]) to conduct structural equation modelling (SEM) to test a model of somatisation associated with PTSD and DSO. SEM is a statistical procedure combining confirmatory factor analysis (CFA) and path analysis. It is a multivariate statistical method that allows for the development of latent variables and an assessment of the direct and indirect associations between these variables.

CFA and SEM model fit were assessed using the chi-square model fit test, comparative fit index (CFI) [37], Tucker-Lewis Index (TLI) [38], root mean square error of approximation (RMSEA) [39], and the Standardised Root-Mean-Square Residual (SRMR) [40]. As per standard recommendations [41], acceptable model fit is indicated by a non-significant chi-square result, CFI and TLI values >0.90, and RMSEA and SRMR values <0.08.

We followed the two-step approach to SEM [42]. Measurement models for each latent variable were first calculated to accurately measure each latent variable in the path analysis. A latent variable representing PTSD was created from the ITQ items measuring the symptoms clusters of re-experiencing, avoidance and a sense of threat. A latent variable representing DSO was created from the ITQ items measuring the symptom clusters of negative self-concept, disturbed relationships and affect dysregulation. Following this, path analysis was conducted to measure the association between the different variables in the model. Path analysis was a direct effects only model of the latent variables PTSD and DSO on PHQ-15 somatisation and included the following variables age, gender, and LEC total score as controls. Controlling for life events allows for further isolating and examining the unique contributions of PTSD and DSO symptoms to explain presence of somatic symptoms.

To appropriately estimate the model despite missing data (29.7% of cases), the maximum likelihood estimation with robust standard errors (MLR) was used (bootstrapping - 5000 iterations). Full information maximum likelihood (FIML) models include missing data within the model, ensuring more accurate estimation.

## 3 Results

Of our 222 participant sample, 176 participants completed all items of the PHQ-15, with between 191 and 217 individuals completing each item. Missing data was accounted for by using an FIML model to conduct path analysis. Demographic characteristics are presented in Table 1.

**Table 1:** Participant demographic variables across groups.

Demographics			
Variable	PTSD (33)	CPTSD (111)	No PTSD (78)
Mean age at time of assessment (SD)	51 (13)	47 (12)	49 (14)

Gender			
Male (%)	19 (58)	55 (50.5)	39 (50)
Female (%)	14 (42)	56 (49.5)	39 (50)

Ethnicity (%)			
White	31 (97)	102 (93)	76 (97)
Non-white	1 (3)	8 (7)	2 (3)

Employment			
Unemployed	20 (63)	77 (70)	40 (51)
Employed	12 (37)	33 (30)	38 (49)

Living arrangements (%)			
Married or cohabiting	16 (50)	48 (44)	48 (62)
Single, widowed, divorced or separated	16 (50)	60 (56)	30 (38)

Higher education: A levels and above (%)			
Higher education	11 (41)	30 (30)	21 (30)
No higher education	16 (59)	69 (70)	50 (70)

Total ITQ mean (SD)	26.30 (6.90)	37.63 (5.68)	18.70 (10.22)
Core PTSD ITQ mean (SD)	16.88 (3.84)	18.87 (3.33)	8.26 (4.58)
DSO ITQ mean (SD)	9.42 (3.61)	18.74 (3.29)	10.45 (6.82)
Total LEC mean (SD)	6.71 (3.66)	7.35 (3.53)	6.21 (3.82)

PTSD = post-traumatic stress disorder, CPTSD = complex post-traumatic stress disorder, ITQ = The International Trauma Questionnaire.

Categorical PHQ-15 somatic symptom severity stratified by probable ICD-11 PTSD diagnosis is shown in Table 2. The CPTSD group reported significantly higher rates of high somatic symptom severity than the no PTSD or PTSD groups (chi-square 33.0, df = 6, *p*-value <0.001). The mean PHQ-15 score for the no PTSD group was 11.0 (SD: 5.5), the PTSD group was 14.7 (SD: 5.7) and the CPTSD group was 17.1 (SD: 6.0). A one-way between groups ANOVA confirmed a statistically significant effect for the No PTSD, PTSD and CPTSD group on PHQ-15 score (F 21.24, df = 2, *p*-value <0.001). A post-hoc Tukey test demonstrated significant variation between the No PTSD and the PTSD group (*p*-value <0.05) and the No PTSD and the CPTSD group (*p*-value <0.001). The variation between the PTSD and the CPTSD group was not significant (*p*-value 0.155).

**Table 2:** Frequency of PHQ-15 somatic symptom severity stratified by probable PTSD/CPTSD diagnosis-.

PHQ-15 Symptom Severity	No PTSD (*n* = 68)	PTSD (*n* = 25)	CPTSD (*n* = 83)
Minimal (%)	10 (15)	0 (0)	3 (4)
Low (%)	15 (22)	4 (16)	4 (5)
Medium (%)	24 (35)	9 (36)	18 (22)
High (%)	19 (28)	12 (48)	58 (70)

PHQ-15 = patient health questionnaire 15 somatic symptom severity scale, PTSD = post-traumatic stress disorder, CPTSD = complex post-traumatic stress disorder.

### 3.1 Path analysis

We used a direct paths model to determine fit. The standardised regression coefficients for direct effects with standard errors and significance values are presented in Table 3. The model chi-square test demonstrated that our model was significantly different to the observed data (chi square 411.6, *p*-value <0.001), however, this should not lead to the rejection of the model as Tanaka [43] has shown that the chi-square test is susceptible to Type 1 errors. The CFI (0.909), TLI (0.898), SRMR (0.114) and the RMSEA (0.062), however, indicated good model fit. The model explained 45.9% of the variance in PHQ-15 score. Contradicting our hypothesis, ITQ PTSD severity, but not ITQ DSO severity, was significantly associated with subsequent PHQ-15 somatisation score. A post-hoc one way between groups ANOVA demonstrated that the CPTSD group had greater core PTSD symptom severity than that of the PTSD group (F = 173.12, df = 2, *p*-value ≤0.001).

**Table 3:** Direct standardised regression coefficients with standard errors and significance levels.

Direct associations	β	SE	*p*-value
PTSD → PHQ-15	0.616	0.259	0.017
DSO → PHQ-15	−0.012	0.255	0.962

All direct regression effects are adjusted for gender, age and total Life Events Checklist score. PTSD = post-traumatic stress disorder, DSO = disturbances in self-organisation, β = standardised regression coefficient, SE = standard error, *p*-value = statistical significance.

## 4 Discussion

We found that people with CPTSD had a higher prevalence of co-morbid somatisation and worse somatisation symptom severity than those with PTSD and those with no PTSD, as measured using the PHQ-15. The model explained 45.9% of the variance in PHQ-15 score, demonstrating the large association of PTSD and CPTSD with somatisation.

The direct effects standardised regression coefficients for path analysis suggest that the most important feature surrounding the development of somatisation in those with PTSD and CPTSD are the core PTSD symptoms themselves. This may be due to higher core PTSD symptom severity in the CPTSD group than in the PTSD group, although due to the qualitative differences between PTSD and CPTSD [[3], [4], [5]] severity alone may not be the complete explanation. It is also likely that these core features are associated with somatisation through a complex interplay of the biological and psychological factors. PTSD alters both systemic immune response [44] and causes behavioural and cognitive changes (post-traumatic cognitions) such as negative attentional bias [12]. Thus, the relationship between PTSD and somatic symptoms is likely to be bidirectional and mutually maintaining [45]. This model of mutual maintenance suggests that PTSD and physical symptoms become conditioned at the time of trauma and that symptoms of each aggravate symptoms of the other [12]. Furthermore, shared vulnerability factors leave some individuals more likely to develop co-occuring somatisation (in particular chronic pain) and PTSD [46]. People with PTSD may also be particularly preoccupied with their somatic complaints. This could be due to hyperarousal and anxiety leading to a catastrophic interpretation of the symptoms, or perhaps an attentional bias towards negative symptoms [47].

PTSD core symptoms seem to be more strongly associated with somatisation than DSO symptoms, and the existing literature highlights the following factors. Research demonstrates that people with PTSD have high utilisation of medical services [48,49]. While physical health problems can be caused by the traumatic event(s) itself, such as war or accidents [48], traumatic stress reactions themselves may cause long term shifts in psychobiology and systemic immune functioning with prolonged physical health complaints as a result of continued hyperarousal [44]. Dysfunctional and unhealthy coping strategies, such as alcohol and drug abuse, as well as comorbid psychiatric diagnoses, perpetuate poor physical health and can increase help seeking [50]. Help seeking via a physical health complaint may also be more acceptable for some people who are reluctant to disclose their psychiatric symptoms due to stigma or because of the characteristic avoidance of PTSD. It is possible that many of these factors are more likely in multiple early trauma exposure, which we might expect in CPTSD [19].

While this study alone cannot determine causation between CPTSD and somatisation, we know that PTSD increases the subsequent risk of somatisation [10]. One study found little evidence that the risk of new PTSD is increased in those with pre-existing somatisation [51], although this may not be the case for everyone. Our path analysis, however, supports this view, with the latent variable of PTSD significantly associated with subsequent somatisation (although the cross-sectional nature of this study limits our ability to demonstrate causation). Thus, PTSD may precede the development of somatisation. DSO, however, did not significantly predict somatisation, a surprising finding considering that people with CPTSD in our cohort have more frequent and severe rates of somatisation than those with PTSD or no PTSD. This could be due to individuals with CPTSD having more severe core symptoms of PTSD which then predict more severe somatisation. These more severe PTSD symptoms may have resulted from multiple childhood traumas, some of which may have been interpersonal, life threatening and characterised by greater peritraumatic distress and dissociation. These factors all predict subsequent PTSD onset, severity and maintenance [52]. It is also possible that our results are subject to residual confounding, with variables we did not assess mediating the relationship, such as functional impairment, depression, anxiety [53]. Furthermore, our unidirectional model only offers a partial explanation for the relationship between PTSD/CPTSD and somatisation.

In our analyses, the rates of somatisation were similar in the PTSD group and the no PTSD group, despite previously documented higher rates of somatisation in patients with PTSD [10]. While many in the trauma-exposed sample did not meet ICD-11 PTSD criteria, all had reported traumatic stress symptoms previously and some had previously been diagnosed with PTSD. This makes it likely that they would report higher symptom levels than a general population control group and possibly account for the fact that a large proportion scored in the clinical range on the measure. Thus, while the no PTSD group represents a traumatised control which leaves comparison with the PTSD and CPTSD groups problematic, the PTSD group allows good comparison with the CPTSD group. This study used a reasonably sized sample (*n* = 222) which is much larger than previous work [19] and used well validated measures to assess frequency, severity and conduct path analysis. Although the demographics were broadly comparable across groups, as displayed in Tables 1, 94% of the sample were caucasian and 62% were unemployed, raising questions about the generalisability of these findings. Furthermore, those with CPTSD fared slightly worse on certain social outcomes, with higher rates of unemployment and single living due to being divorced, separated or widowed. This is comparable to previous demographic findings investigating DESNOS [54], and seems expected considering the disturbed relationships requirement for a diagnosis of ICD-11 CPTSD. The study also relied on self-report measures and as such we were only able to make a probable diagnosis of CPTSD/PTSD/somatic symptom severity. The accuracy of ITQ rates of PTSD/CPTSD are not known, and, at present, there is no gold standard interview measure for ICD-11 PTSD/CPTSD, although this is currently being developed. The sample had a large proportion of missing PHQ-15 data (29.7% of cases), some of which was related to male participants not responding to an item about menstruation. Furthermore, the PHQ-15 assesses physical symptoms generally, some of which may be due to pre-existing physical comorbidity, rather than somatisation [33]. Future research should replicate our findings and further explore the underlying etiological mechanisms that may contribute to the increased somatic symptom burden in those with CPTSD.

This study is the first to demonstrate higher rates and greater severity of somatisation in those with ICD-11 CPTSD than in those with PTSD. Path analysis suggests that it is the core symptoms of PTSD, not DSO which are associated with the development of somatisation. The underlying aetiology is likely to represent a complex interplay of biological, psychological and social factors which cause and then maintain bodily distress. Importantly, our findings suggest that clinicians should consider this high somatic symptom burden in those with CPTSD during assessment and treatment.

## Funding

This work was supported by the MRC Clinical Academic Mentorship Scheme to LAW.

## Declaration of interests

We declare no competing interests.

## Data availability statement

The data that support the findings of this study are available on request from the corresponding author. The data are not publicly available due to privacy or ethical restrictions.
